# Combination of Genome-Scale Models and Bioreactor Dynamics to Optimize the Production of Commodity Chemicals

**DOI:** 10.3389/fmolb.2022.855735

**Published:** 2022-04-27

**Authors:** Jorge Lázaro, Giorgio Jansen, Yiheng Yang, Mario A. Torres-Acosta, Gary Lye, Stephen G. Oliver, Jorge Júlvez

**Affiliations:** ^1^ Department of Computer Science and Systems Engineering, University of Zaragoza, Zaragoza, Spain; ^2^ Cambridge Systems Biology Centre and Department of Biochemistry, University of Cambridge, Cambridge, United Kingdom; ^3^ Department of Biochemical Engineering, University College London, London, United Kingdom

**Keywords:** flexible nets, modeling formalisms, model integration, multi-scale models, genome-scale models, commodity chemicals, continuous culture, citramalate production

## Abstract

The current production of a number of commodity chemicals relies on the exploitation of fossil fuels and hence has an irreversible impact on the environment. Biotechnological processes offer an attractive alternative by enabling the manufacturing of chemicals by genetically modified microorganisms. However, this alternative approach poses some important technical challenges that must be tackled to make it competitive. On the one hand, the design of biotechnological processes is based on trial-and-error approaches, which are not only costly in terms of time and money, but also result in suboptimal designs. On the other hand, the manufacturing of chemicals by biological processes is almost exclusively carried out by batch or fed-batch cultures. Given that batch cultures are expensive and not easy to scale, technical means must be developed to make continuous cultures feasible and efficient. In order to address these challenges, we have developed a mathematical model able to integrate in a single model both the genome-scale metabolic model for the organism synthesizing the chemical of interest and the dynamics of the bioreactor in which the organism is cultured. Such a model is based on the use of Flexible Nets, a modeling formalism for dynamical systems. The integration of a microscopic (organism) and a macroscopic (bioreactor) model in a single net provides an overall view of the whole system and opens the door to global optimizations. As a case study, the production of citramalate with respect to the substrate consumed by *E. coli* is modeled, simulated and optimized in order to find the maximum productivity in a steady-state continuous culture. The predicted computational results were consistent with the wet lab experiments.

## 1 Introduction

Methyl methacrylate (MMA) is a volatile synthetic chemical used mainly in the preparation of acrylic emulsion and extrusion resins. Polymers and co-polymers containing methyl methacrylate are used as solvents, adhesives, sealants, leather and paper coatings, inks, textiles, dental prothesis, etc.

There are 17 different routes widely used in industry that end up synthesizing MMA. The main problem with these routes is that all the precursor molecules (ethylene, propyne, propylene, tert-butyl alcohol, isobutene and isobutane) have their origin in non-renewable sources such as petroleum and natural gas whose extraction is highly damaging for ecosystems (Sugiyama et al., 2009).

An alternative approach consists of considering citramalate, a precursor for the synthesis of MMA, which is produced by *Methanocaldococcus jannaschii*. The production of citramalate in *M. jannaschii* is due to the presence of the gene *cimA* which encodes the enzyme citramalate synthase (EC: 2.3.1.182). This enzyme catalyses the reaction in which one molecule of acetyl-CoA, one molecule of pyruvate and one molecule of water react to produce one molecule of (3 R)-citramalate, one molecule of CoA and liberating a proton (UniProt Consortium, 2019):

$$acetyl-CoA+pyruvate+H_{2}O\to \left(3R\right)citramalate+CoA+H^{+}$$ (1)

One of the most difficult tasks when carrying out the design of a biological experiment is setting the conditions and parameters that have to be tracked during the experiment. Computational models can help overcome these difficulties by providing the researchers with guidance when designing experiments in the wet lab, thus avoiding costly trial-and-error approaches.

In Webb et al. (2018), researchers could reach an efficient bioproduction of citramalic acid by a genetically engineered *E. coli* strain which included the gene *CimA*. The fact that the cell culture operated in fed-batch mode suggests that the production could be optimized by changing to continuous culture. In a continuous culture, a steady state is reached when the macroscopic variables of the tank remain constant over time. The complexity of continuous cultures lies in the fact that identical macroscopic conditions may trigger multiple steady states. The potential steady states of an *E. coli* continuous culture are characterized in Fernandez-de Cossio-Diaz et al. (2017).

Flux Balance Analysis (FBA) Orth et al. (2010) has proven to be an extremely useful approach to analyze steady states of genome-scale constraint-based models. FBA assumes the attainment of a steady state of intracellular metabolite concentrations to compute reactions fluxes by means of a linear programming problem. In addition to analyzing the potential steady states, a challenging problem when modeling and optimizing a continuous culture consists of linking the microscopic variables of the genome-scale model with the macroscopic variables of the bioreactor, e.g., metabolite concentrations out of the cells.

A variation of the FBA approach, called *Dynamic Flux Balance Analysis* (DFBA) (Mahadevan et al., 2002a), can be used to couple intracellular metabolism with the dynamics of the extracellular metabolite concentrations. DFBA has been applied for the production of several metabolites. In Flassig et al. (2016), the production of *β*-carotene in green microalgae was optimized for a fed-batch continuous culture. The work in Hanly et al. (2013) validated and optimized a yeast dynamic flux balance model in order to determine the optimum conditions that maximize the production of ethanol in a batch culture of *S. cerevisiae*. Two approaches to predict batch growth of *E. coli* based on DFBA are introduced in Mahadevan et al. (2002b). DFBA was applied as well in (Meadows et al. (2010)) with the aim of simulating simultaneous acetate and glucose consumption and evaluate the behaviour of *E. coli* cells in different types of media. Although DFBA has been applied successfully in many areas, it has some limitations as it assumes quasi-steady-state conditions (Reimers and Reimers,^2016^) and has been used almost exclusively on batch and fed-batch cultures.

Other methods not based on DFBA, such as k-OptForce, have been used to integrate kinetics in constraint-based models. For instance, the optimization of the production of L-serine in mutant *E. coli* and triacetic acid lactone in mutant *S. cerevisae* were performed in Chowdhury et al. (2014). K-OptForce uses kinetic rate expressions to redistribute fluxes in the metabolic network, instead of relying on surrogate fitness functions such as biomass maximization. For additional information on this topic, a review of efforts to integrate kinetic information in constraint-based models can be found in Kim et al. (2018).

In contrast to the previous works, we propose the use of Flexible Nets (FNs) Júlvez et al., 2018), a modeling framework that produces analytical models that can be represented graphically and that are well suited for analysis and optimization, in order to design an overall computational model that combines both the bioreactor dynamics and the metabolic network of the cultured organism. In addition to facilitating the integration of a macroscopic and a microscopic model, FNs can accommodate uncertain parameters and can approximate non-linear dynamics. In particular, constraint-based models Sigmarsdóttir et al. (2020) of metabolic networks can be straightforwardly mimicked and analyzed by FNs. Notice that such models do not account for the concentration of species, and loose flux bounds are usually associated with the reactions. Moreover, FNs can also model the differential equations that determine the dynamics of the bioreactor variables, e.g., cell density, nutrient supply, and metabolite concentration. In this way, FNs can integrate, in a seamless model, both the genome-scale model of the cultured microorganism and the bioreactor dynamics. We show how such an integrated model can be developed and exemplify the process through the modeling and simulation of a system that produces citramalate by a genetically modified *E. coli* culture. The optimization of the model obtained with respect to citramalate productivity provides the optimal settings, i.e., intracellular fluxes and bioreactor parameters, that maximize the productivity of citramalate in a steady-state continuous culture.

## 2 Materials and Methods

### 2.1 Flexible Nets

Flexible Nets (FNs) is a modeling formalism for dynamic systems inspired by Petri Nets, see Murata (1989); Silva, 1993) for a gentle introduction. FNs aim to capture the relationship between the state and the processes of a given dynamic system by means of two interconnected nets: the event net and the intensity net. On the one hand, the event net models how the processes modify the state variables. On the other hand, the intensity net models how the state variables determine the speeds of the processes. In contrast to Petri nets, both the event and the intensity nets are tripartite graphs which have three types of vertices: places, transitions and handlers. The handlers of the event net are called event handlers, and the handlers of the intensity net are called intensity handlers. Places (which are depicted as circles) are associated with metabolites and transitions (which are depicted as rectangles) are associated with reactions. Event handlers (which are depicted as dots) capture the change of concentration of metabolites produced by reactions. Intensity handlers (also depicted as dots) model how the concentrations of metabolites determine the speeds of reactions. Although event and intensity handlers can be distinguished by the net elements to which they are connected, for clarity the arcs and edges of event handlers will be drawn in black and those of intensity handlers in blue.

**FIGURE 1:**
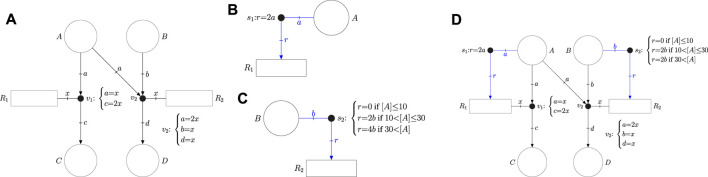
**(A)** Event net modeling the stoichiometry of reactions *R*_1_: *A* →2 *C* and *R*_2_2*A*+ *B* → *D*. **(B**) Intensity net producing a speed in *R*_1_ proportional to the concentration [*A*]. **(C)** Guarded intensity net producing a speed in *R*_2_ equal to 0 if [*A*] is below 10, 2 [*B*] if [*A*] is between 10 and 30, and 4 [*B*] otherwise. **(D)** Flexible net combining the event net in **(A)** and the intensity nets in **(B)** and **(C)**.

As an example, the event net in Figure 1A has four places {*A*, *B*, *C*, *D*}, two reactions {*R*_1_, *R*_2_} and two event handlers {*v*_1_, *v*_2_}. Such a net models the following reactions:

$$\begin{array}{ll} R_{1}:\; & A\to 2\;C\\ R_{2}:\; & 2A+B\to D \end{array}$$

The stoichiometry of the reactions is modeled by the equalities associated with the event handlers. In particular, the equalities *a* = *x* and *c* = 2*x* of *v*_1_ imply that each occurrence of reaction *R*_1_ consumes one unit of metabolite *A* and produces two units of metabolite *C* (such units usually refer to concentrations). On the other hand, the equalities *a* = 2*x*, *b* = *x*, and *d* = *x* of *v*_2_ mean that each occurrence of *R*_2_ consumes 2 units of *A*, 1 unit of *B* and produces 1 unit of *D*.

The event net in Figure 1A does not establish any dynamics, it just models the stoichiometry. The dynamics of reactions can be specified by the intensity net. For instance, the intensity net in Figure 1B specifies the speed of reaction *R*_1_ as twice that of the concentration of *A*, see equation *r* = 2*a* associated with the intensity handler *s*_1_. In addition to equalities, intensity handlers can be associated with inequalites to model uncertainty, e.g., if 1.8*a* ≤ *r* ≤ 2.2*a* was associated with *s*_1_ then the speed of *R*_1_ could be any value in the interval [1.8 [*A*], 2.2 [*A*]] where [*A*] is the concentration of *A*.

Moreover, several sets of equalities and inequalities can be associated with the same intensity handler. If this is the case, the set of inequalities that rules the reaction dynamics is determined by the concentrations of the system. The intensity net in Figure 1C associates three different equalities with the intensity handler *s*_2_ which imply that the speed of *R*_2_ is 0 if [*A*] is below 10, 2 [*B*] if [*A*] is between 10 and 30, and 4 [*B*] otherwise. Intensity handler *s*_2_ is said to be guarded, it has three guards (or regions) that can determine the speed of *R*_2_. Guarded handlers can be exploited to approximate non-linear kinetics of reactions.

The event net (Figure 1A) can be combined with the intensity nets (Figure 1B and Figure 1C) to produce an FN (Figure 1D) which models both the stoichiometry and dynamics of the system.

### 2.2 FNs to Model Constraint-Based Models

A *constraint-based model* (Varma and Palsson, 1994) can be expressed as a tuple {R,M,S,L,U} where R is the set of reactions, M is the set of metabolites, $S\in R^{\left|M|\times |R\right|}$ is the stoichiometric matrix, and $L,U\in R^{\left|R\right|}$ are lower and upper flux bounds of the reactions (notice that very loose flux bounds can be assigned when no kinetic information is available). The concentrations of metabolites are usually disregarded in constraint-based models, being the main focus of most analyses on the fluxes of reactions. This section shows how constraint-based models can be expressed graphically and analyzed numerically in a straightforward way by FNs.

**FIGURE 2:**
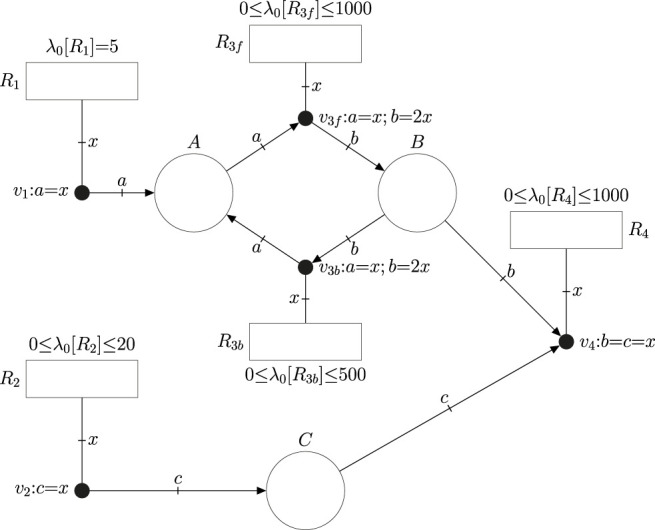
FN modeling the constraint based model expressed by the reactions in Table 1. The flux bounds of the reactions are modeled by the default intensities (or speeds) *λ*_0_ of transitions. Reversible reactions like *R*_3_ are unfolded into a forward and a backward reaction with non-negative flux.

Consider the constraint-based model defined by Table 1. It consists of four reactions together with their corresponding flux bounds. The FN in Figure 2 models such a constraint-based model. The net has one place per metabolite, one transition per reaction, and one event handler per reaction. The equalities associated with the event handlers model the stoichiometry of the reactions. Since constraint-based models do not account for the concentrations of metabolites, the fluxes of reactions cannot depend on concentrations. Hence, the corresponding FN does not have intensity handlers. The range of potential fluxes of reactions is modeled by a parameter *λ*_0_ that is associated with each transition, e.g. *λ*_0_ [*R*_1_] = 5 in *R*_1_ means that the flux of *R*_1_ will always be equal to 5 *mmol gDW* ^−1^ *h* ^−1^, and 0 ≤ *λ*_0_ [*R*_2_] ≤ 20 in *R*_2_ implies that the flux of *R*_2_ can be any quantity between 0 and 20 *mmol gDW* ^−1^ *h* ^−1^.

**TABLE 1:** Simple constraint-based model of four reactions with lower and upper flux bounds (*mmol gDW* ^−1^ *h* ^−1^). The model is represented graphically by the FN in Figure 2

Reaction	Lower bound	Upper bound
*R*_1_: ∅ → *A*	5	5
*R*_2_: ∅ → *C*	0	20
*R*_3_: *A* ↔ 2*B*	−500	1,000
*R*_4_: *B*+ *C* → ∅	0	1,000

The fluxes of transitions must be non-negative in FNs. Thus, the modeling of reversible reactions like *R*_3_ requires its unfolding into two reactions, a forward reaction *R*_3*f*_ and a backward reaction *R*_3*b*_ with appropriate limits for their parameters *λ*_0_ (Júlvez and Oliver, 2020b). If no *λ*_0_ is explicitly associated with a reaction *r*, then it is assumed that *λ*_0_ [*r*] = 0. In general, the speed of *r* is equal to *λ*_0_ [*r*] plus the intensities provided by the intensity handlers to which it is connected (see Figure 1B).

FNs can be analyzed by building a set of mathematical constraints that the state of the system necessarily satisfies (Júlvez et al., 2018). The association of such constraints with an objective function of interest results in a programming problem whose solution yields a theoretical optimum. For instance, if the objective function for the FN in Figure 2 is the maximization of the flux of *R*_4_ in the steady state, then the solution of the programming problem would be 10 *mmol gDW* ^−1^ *h* ^−1^ which is the theoretical maximum steady-state flux of *R*_4_. In addition to the flux of *R*_4_, fluxes for the rest of reactions are obtained. In this particular case, this approach is equivalent to performing Flux Balance Analysis (FBA) Orth et al. (2010) on the constraint-based model.

For the production of citramalate, the constraint-based model of the organism *Escherichia coli* strain K-12 MG1655 (Webb et al. (2018) was considered. The model is named iJO1366 in the BiGG repository database (Orth et al. (2010)) and has 1805 metabolites, 2,583 reactions, and 1,367 genes. The reaction in Eq. 1 was added to this model, simulating a transgenic *E. coli* strain capable of synthesizing citramalate.

The transformation of the resulting constraint-based model into an FN can be carried out by following the approach to obtain the net in Figure 2. Such an approach is performed automatically by the *cobra2fn* module of the *Python* tool *fnyzer* (Júlvez and Oliver, 2020a).

### 2.3 FNs to Model Bioreactor Dynamics

**FIGURE 3:**
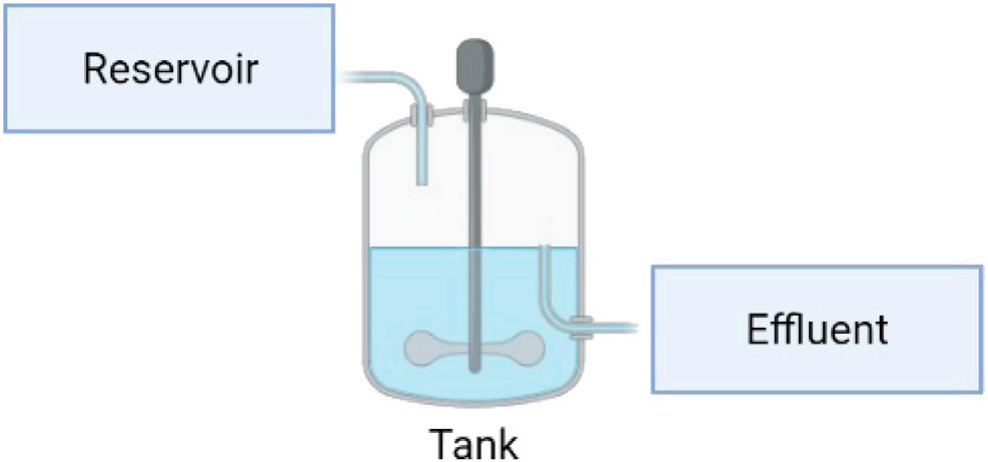
Sketch of a bioreactor in continuous culture mode. The nutrient supply and the removal of toxic and cell products are executed at the same time uninterruptedly. The three main compartments are: reservoir, tank and effluent.

The macroscopic model of the bioreactor consists of three parts (see Figure 3) the “Reservoir”, which contains the fresh sterile medium and supplies the cell culture with the essential nutrients for cell survival; the “Tank”, where the cell culture is placed, and the “Effluent” which clears away the accumulated products and some of the cells in the tank.

The dynamics of the bioreactor variables, which are named macroscopic variables, are determined by differential equations (Fernandez-de Cossio-Diaz et al., 2017). The equation that expresses the evolution of the cell density in the tank is:

$$\frac{dX}{dt}=\left(\mu -D\right)X$$ (2)

where *X* (*gDWL* ^−1^) is the cell density in the tank, *μ* is the effective cell growth rate (*h* ^−1^), and *D* is the dilution rate (*h* ^−1^), which is the rate at which culture fluid is replaced divided by the culture volume.

The evolution of the concentration of a given metabolite, *i*, in the tank is given by:

$$\frac{ds_{i}}{dt}=\left(c_{i}-s_{i}\right)D-u_{i}X$$ (3)

where *c*_*i*_ is the concentration of the metabolite *s* in the medium (*mM*), *s*_*i*_ is the concentration of the metabolite in the tank (*mM*), *D* is the dilution rate (*h* ^−1^), *u*_*i*_ is the specific uptake rate of the metabolite by the cells (*mmol gDW* ^−1^ *h* ^−1^), and *X* is the cell density in the tank (*gDW L* ^−1^). If *u*_*i*_ > 0 the metabolite is consumed by the cell, otherwise (*u*_*i*_ < 0) the metabolite is secreted from the cell.

For the particular case of a system in which glucose, denoted as metabolite *g*, is consumed by an *E. coli* culture, Eq. 3 for the concentration of glucose in the tank becomes:

$$\frac{ds_{g}}{dt}=\left(c_{g}-s_{g}\right)D-u_{g}X$$ (4)

where *c*_*g*_ is the concentration of glucose in the supply medium, *s*_*g*_ is the concentration of glucose in the tank and, *u*_*g*_ is the glucose uptake flux by the cell (which is a positive value).

The variation of a given product, e.g., citramalate, denoted as *c*, is derived from Eq. 3 as:

$$\frac{ds_{c}}{dt}=-s_{c}D-u_{c}X$$ (5)

where *s*_*c*_ is the citramalate concentration in the tank, and *u*_*c*_ is the citramalate secretion flux. Notice that, since citramalate is secreted from the cell, *u*_*c*_ is negative, and hence, − *u*_*c*_ *X* is a positive contribution of citramalate to the tank.

**FIGURE 4:**
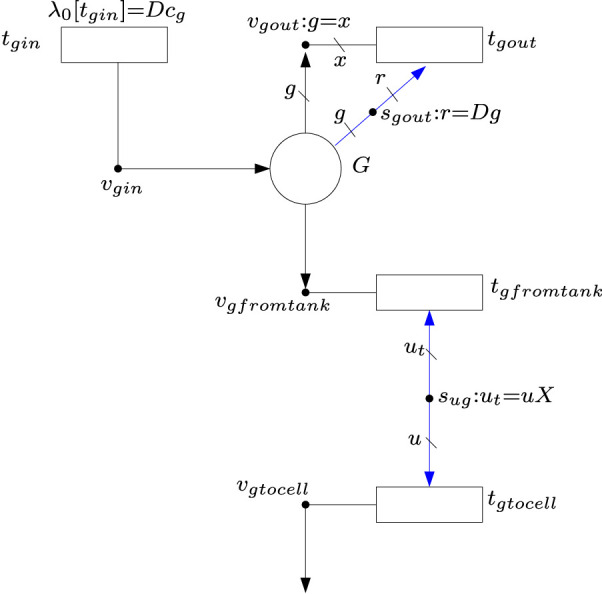
FN modeling the differential Eq. 4

The above differential equations can be modeled by FNs.^1^ For instance, Eq. 4 can be modeled by the FN in Figure 4 where place *G* accounts of the concentration of glucose in the tank. As established by Eq. 4, place *G* has one input flux and two output fluxes. The input flux comes from the reservoir, it is modeled by transition *t*_*gin*_, and it is equal to *D* ⋅ *c*_*g*_. As this is a constant amount, no intensity handlers are needed, and the flux is modeled by the *λ*_0_ associated with *t*_*gin*_. The output flux modeled by *t*_*gfromtank*_ represents the uptake rate of glucose by the cell culture, and it is equal to *u*_*g*_ *X* where *u*_*g*_ is the specific uptake rate and *X* is the cell biomass. This flux is produced by the intensity handler *s*_*ug*_. Such an intensity handler scales by *X* the amount of glucose that is consumed by the cells, see equation *u*_*t*_ = *uX* associated with *s*_*ug*_. The output flux modeled by *t*_*gout*_ represents the glucose that leaves the tank without being captured by the cells. Such an output flux is equal to the dilution rate times the concentration of glucose in the tank, see equation *Ds*_*g*_ associated with *s*_*gout*_: *r* = *Ds*_*g*_.

**FIGURE 5:**
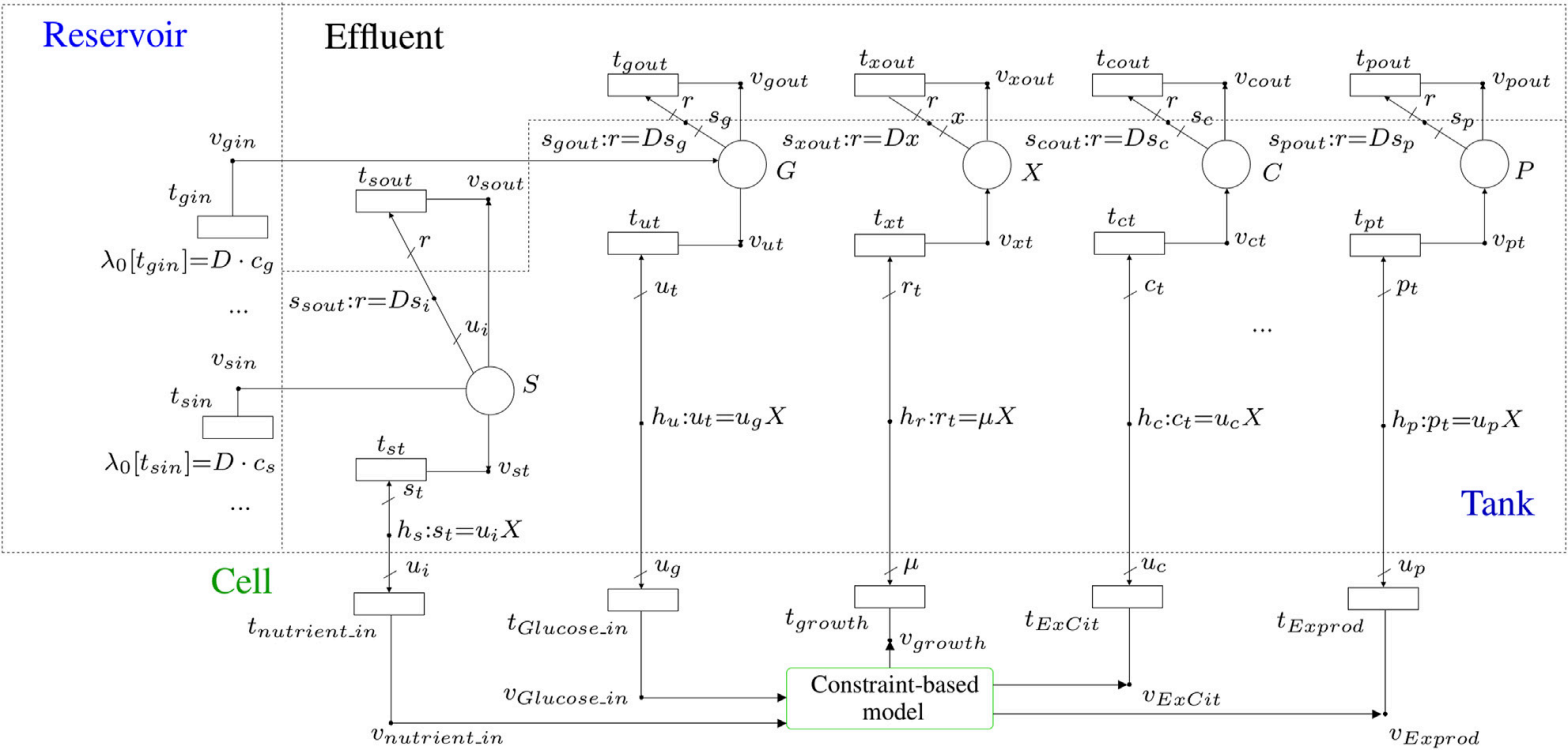
FN modeling the overall production system by integrating the macroscopic model of the bioreactor and the microscopic model of the constrained-based model, MODEL1108160000 Orth et al. (2011) of the BioModels database Malik-Sheriff et al. (2020), of the cultured organism.

Eq. 5 can be modeled similarly by FNs, see place *C* and the elements connected to it in Figure 5. In this case, there is one input flux and one output flux. The input flux comes from the cell (citramalate is produced by the culture), it is modeled by transition *t*_*ct*_ and it is equal to *u*_*c*_ *X*, see equation associated with *h*_*c*_. The output flux corresponds to the amount of citramalate in the tank, once it has been released by the cell, that forms part of the effluent. Such a flux is modeled by *t*_*cout*_ and it is equal to *s*_*c*_ *D*, see equation associated with *s*_*cout*_.

Finally, Eq. 2 is modeled by the place *X* and the net elements connected to it. The input flux of *X*, i.e. the rate at which *X* increases, is modeled by *t*_*xt*_ and it is equal to the specific growth rate of the culture times the cell density, *rX*, see equation associated with *h*_*r*_. The output flux of *X*, i.e. the rate at which *X* decreases, corresponds to the cells that are cleared away in the continuous culture, it is modeled by *t*_*xout*_ and is equal to *Dx*, see equation associated with *s*_*gout*_.

### 2.4 Model Integration

The merger of the FNs that model the dynamics of metabolite concentrations in the bioreactor, see Section 2.3, and the FNs that model the constraint-based model of the metabolic network of the cultured organism, see Section 2.2, results in a single FN that models the overall production system, see Figure 5. The nutrients in the medium can be introduced by the transitions located in the reservoir compartment. In this compartment, as many transitions as metabolites in the medium are required. For example, transition *t*_*gin*_ accounts for the presence of glucose in the medium, and transition *t*_sin_ represents the presence of a given metabolite *s* in the medium.

The concentrations of metabolites in the bioreactor are modeled by the places in the tank compartment. In a general system, there will be as many places in the tank compartment as there are metabolites being tracked. In Figure 5, place *S* represents a generic nutrient, i.e., it is provided by the medium, it is consumed by the culture (see arrow going from *S* to *v*_*st*_) and forms part of the effluent. On the other hand, place *P* represents a generic product, i.e., it is not provided by the medium, it is produced by the culture (see arrow going from *S* to *v*_*st*_) and forms part of the effluent. Although the cell density, *X*, is not a metabolite, its evolution, see Eq. 2, can be modeled exactly as it was a product of the culture, see place *X* and the net elements connected to it (the increase of *X* is due to the biomass production which is determined by the growth rate).

For the particular system that produces citramalate by a genetically modified *E. coli* culture, glucose-limited conditions are assumed. Thus, in addition to the cell density modeled by place *X* with units *gDWL* ^−1^, the focus will be on the concentration of one nutrient (glucose which is modeled by place *G* with units *mM*), and the concentration of one product (citramalate which is modeled by place *C* with units *mM*). Recall that the dynamics of *X* is ruled by Eq. 2, while *G* and *C* are ruled by Eqs 4 and 5, respectively. The microscopic model, i.e., constrained-based model, for the citramalate production system is given by the genome-scale metabolic model of *E. coli* strain K-12 (iJO1366), MODEL1108160000 Orth et al. (2011) of the BioModels database (Malik-Sheriff et al., 2020). This model was converted to an FN by the *fnyzer* tool (Júlvez and Oliver, 2020a).

The integration of the macroscopic and the microscopic models was possible thanks to the elements involved in the interface between the tank and the cell compartments. The main elements taking part in this connection are the intensity handlers *h*_*u*_, *h*_*r*_ and *h*_*c*_. These intensity handlers relate the macroscopic variables of the bioreactor with the exchange fluxes of the cell in such a way that each macroscopic flux equals *X* times the exchange flux of the cell, *X* being the cell density in the tank. The equations which model the interface between the cell and the tank are: *u*_*t*_ = *u*_*g*_ *X*, *r*_*t*_ = *μX*, and *c*_*t*_ = *u*_*c*_ *X*, which are associated with the intensity handlers *h*_*u*_, *h*_*r*_ and *h*_*c*_, respectively.

The aforementioned intensity handlers are graphically located at the interface between the cell and tank compartments, and each one acts as a bridge between two transitions: *h*_*u*_ connects *t*_*Glucose*_*in*_ and *t*_*ut*_, *h*_*r*_ connects *t*_*growth*_ and *t*_*xt*_, and *h*_*c*_ connects *t*_*ExCit*_ and *t*_*ct*_. In our model, which contains all the metabolic reactions of *E. coli* strain K-12 (iJO1366) combined with the reactions that allow citramalate production, *t*_*Glucose*_*in*_ represented the glucose exchange reaction, *t*_*growth*_ is used for the biomass reaction and *t*_*ExCit*_ defined the citramalate exchange reaction.

### 2.5 Model Optimization

This section discusses the approximations that must be applied to the FN in Figure 5 prior to its optimization (Subsection 2.5.1), as well as the type of objective function that is considered (Subsection 2.5.2).

#### 2.5.1 Tackling Non-linearities

Notice that the equations associated with the intensity handlers at the interface between the macroscopic model of the tank and the microscopic model of the cell are not linear. For instance, the equation associated with *h*_*u*_ is *u*_*t*_ = *u*_*g*_ *X* where both *u*_*g*_ (the uptake rate of glucose) and *X* (the cell density) are real variables. The optimization of a non-linear system is, in general, very demanding from a computational point of view. To overcome such a computational burden, non-linear equations can be approximated by piece-wise linear inequalities that are associated with intensity handlers. This approximation results in a guarded FN (see Section 2.1).

A non-linear equation such as *u*_*t*_ = *u*_*g*_ *X* of *h*_*u*_ can be approximated piece-wise linearly by partitioning the state space of one of the real variables, e.g., *X*, into a number of regions and associating a linear inequality with each of the regions. Thus, *h*_*u*_: *u*_*t*_ = *u*_*g*_ *X* can be approximated by:

$$h_{u}:\left\{\begin{array}{cc} X_{min}\cdot u_{g}\leq u_{t}\leq X_{1}\cdot u_{g}\; & \text{if}\;X_{min}\leq X<X_{1}\\ X_{1}\cdot u_{g}\leq u_{t}\leq X_{2}\cdot u_{g}\; & \text{if}\;X_{1}\leq X<X_{2}\\ X_{2}\cdot u_{g}\leq u_{t}\leq X_{3}\cdot u_{g}\; & \text{if}\;X_{2}\leq X<X_{3}\\ \ldots ,\;\\ X_{n-1}\cdot u_{g}\leq u_{t}\leq X_{max}\cdot u_{g}\; & \text{if}\;X_{n-1}\leq X\leq X_{max} \end{array}\right.$$ (6)

where *X*_min_ and *X*_max_ are lower and upper bounds for the cell density, i.e., the cell density is known to be in the interval [*X*_min_, *X*_max_] (notice that these bounds do not need to be tight).

The above approximation considers *n* regions, the first region is active if the cell density *X* is in the interval [*X*_min_, *X*_1_] (in general, the *ith* region is active if the cell density *X* is in the interval [*X*_*i*−1_, *X*_*i*_]). The values *X*_1_, … , *X*_*n*−1_ do not need to be evenly separated, the only condition they must satisfy is *X*_min_ < *X*_1_ < …, < *X*_*n*−1_ < *X*_max_. This way, one and only one region is active at any particular time. The region that is active determines the linear inequality that is used to produce intensity, i.e., if region *i* is active then the intensity produced by *h*_*u*_ can be any value in the interval [*X*_*i*−1_ ⋅ *u*_*g*_, *X*_*i*_ ⋅ *u*_*g*_]. Clearly, the higher the number of regions (and hence, the smaller the regions), the better the approximation to the original non-linear equation. Since a higher number of regions involves a longer run time, there is a trade-off between accuracy and computational cost. As discussed below, the number of regions was determined experimentally so that both the computational burden and the obtained precision are acceptable.

Notice that the previously defined regions can also be used to approximate the non-linear equations of the other handlers in the interface between the macroscopic and microscopic models because all include *X* in their equations. Given that the number of regions has a direct impact on the complexity of the programming problem which needs to be solved (the number of binary variables is linear in the number of regions), partitioning *X* instead of *u*_*g*_ is advantageous from a computational point of view.

**FIGURE 6:**
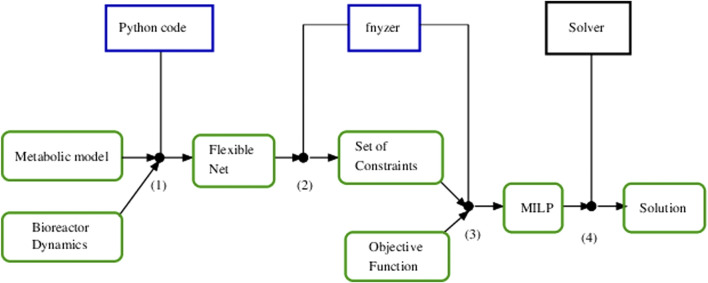
Pipeline showing the steps performed to optimize the integrated model. **(1)** The metabolic model and the bioreactor dynamics are combined to generate a Flexible Net that integrates the macroscopic (dilution rate, substrate concentration, cell density) and microscopic variables (intracellular metabolite fluxes). **(2)** A set of mathematical constraints is derived from the net specification and **(3)** the objective function is selected. The tool fnyzer performs the generation of a mixed-integer linear programming problem according to the set of constraints and the objective function. **(4)** Finally, the MILP problem is easily solved by using a solver (e.g., CPLEX, Gurobi, GLPK) that computes the mathematical solution.

The overall procedure to optimize an FN that integrates a bioreactor and a metabolic network is outlined in Figure 6. After integrating both models in a single FN, a set of mathematical constraints that represent necessary reachability conditions for the state of the system are derived. Such a set of constraints can be derived automatically by the *Python* tool *fnyzer* (Júlvez and Oliver, 2020a). The addition of an objective function to the constraints results in a mixed-integer linear programming (MILP) problem whose solution represents the theoretical optimum state that the system can achieve.

In order to speed up the model optimization, a non-guarded FN has been defined for each of the above regions, and each of these nets has been solved separately. For instance, region *i* determines an FN in which the value of *X* is constrained to the interval [*X*_*i*−1_, *X*_*i*_] (this constraint will be part of the programming problem) and the inequalities *X*_2_ ⋅ *u*_*g*_ ≤ *u*_*t*_ ≤ *X*_3_ ⋅ *u*_*g*_ are associated with *h*_*u*_ (similar inequalities are associated with the rest of handler in the interface). The programming problem associated with each of these nets is linear, and hence, can be solved very efficiently. The optimum solution of the original guarded FN can be obtained straightforwardly by taking the maximum of all the computed objective values of the particular non-guarded FNs.

**FIGURE 7:**
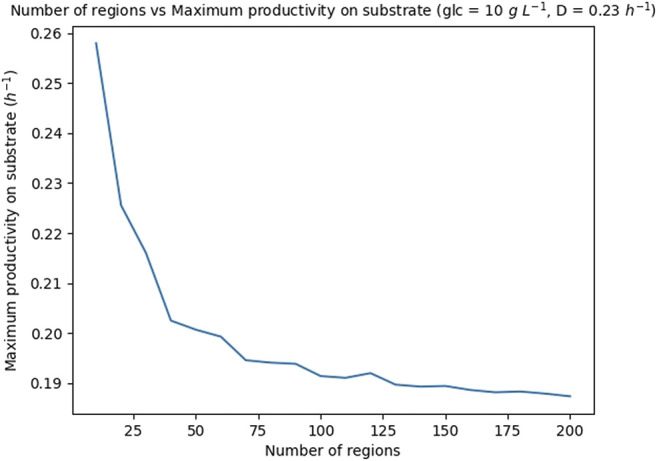
Theoretical maximum productivity on substrate *PS* with respect to the number of regions in which the biomass is partitioned. The glucose concentration is set to 10 *g* *L* ^−1^ and the dilution rate to 0.23 *h* ^−1^.

In order to partition the cell concentration *X* in an appropriate number of regions, the productivity on substrate (*PS*), see Subsection 2.5.2, was calculated repeatedly for different number of regions, ranging in the interval [10, 200], and fixed values of glucose concentration in the medium, 10 *gL* ^−1^, and dilution rate, 0.23 *h* ^−1^. The obtained maximum *PS* are shown in Figure 7 and the CPU run-times are reported in Supplementary Data S3. Notice that after an initial sharp decrease, the productivity converges to a given value. On the other hand, the run-time of the simulations increases linearly with the number of regions (see reported run-times). Based on these results, it was decided to set the number of regions for the optimizations to 100, as this number provided a good trade-off between accuracy and run-time (the run-time to optimize the FN for a given glucose concentration and a given dilution rate is 564 s (9.4 min), see hardware features in Supplementary Data S3.

#### 2.5.2 Optimizing the Productivity

Among the different objective functions that can be considered, we focus on two measures for the productivity of a culture in continuous mode: 1) volumetric productivity, and 2) productivity on substrate.

The volumetric productivity, a. k.a. space-time yield, accounts for the amount of product produced per liter and per hour, it will be denoted *VP* and is expressed in grams of product per liter per hour, i.e., *g* ⋅ *L* ^−1^ ⋅ *h* ^−1^. In terms of the FN in Figure 5, the volumetric productivity corresponds to the outgoing flux of product *flux*(*P*), i.e. intensity of transition *t*_*pout*_, expressed in *g* ⋅ *L* ^−1^ ⋅ *h* ^−1^. The volumetric productivity would be the primary concern of a chemical engineer designing an industrial process, which needs to be both feasible and economically viable.

The productivity on substrate takes into account both the specific growth rate of the culture, *μ* (units *h* ^−1^), and the product yield coefficient, *Y*_*P*/*S*_, where *Y*_*P*/*S*_ denotes the number of grams of product that are produced per Gram of substrate fed into the tank, i.e. $Y_{P/S}=\frac{Product\;\left(g\right)}{Substrate\;\left(g\right)}$ , and hence *Y*_*P*/*S*_ is unitless. In particular, the productivity on substrate, which will be denoted *PS*, for a given net N is defined as:

$$PS\left(N\right)=\mu \cdot Y_{P/S}$$ (7)

given that *Y*_*P*/*S*_ is unitless, PS(N) is expressed in *h* ^−1^. Notice that Eq. 7 entails a trade-off between biomass formation and product production. Although the productivity on substrate is usually a secondary concern, it becomes more relevant as the cost of the substrate increases with respect to the selling price of the product. The productivity on substrate is also a useful metric for a synthetic biologist comparing the performance of different genetically engineered microbial strains.

In the following, the mathematical relation between volumetric productivity and productivity on substrate is explored. In a continuous culture, the yield *Y*_*P*/*S*_ is equal to the grams of product produced per Gram of substrate per time unit, i.e. it can be expressed as $\frac{flux\left(P\right)}{flux\left(S\right)}$ where *flux*(*P*) is the flux of produced product in *g* ⋅ *L* ^−1^ ⋅ *h* ^−1^, and *flux*(*S*) is the flux of provided substrate in *g* ⋅ *L* ^−1^ ⋅ *h* ^−1^. Notice that *flux*(*S*) is equal to *Dc*_*s*_ where *D* is the dilution rate in *h* ^−1^ and *c*_*s*_ is the concentration of the substrate in the fresh medium in *g* ⋅ *L* ^−1^. This way, the yield can be expressed as:

$$Y_{P/S}=\frac{flux\left(P\right)}{flux\left(S\right)}=\frac{flux\left(P\right)}{D\cdot c_{s}}$$ (8)

On the other hand, the cell density is assumed to be constant in a continuous culture, i.e., $\frac{dX}{dt}=0$ , and hence, Eq. 2 implies that *μ* = *D*. Thus, Eq. 7 can be rewritten as:

$$PS\left(N\right)=\mu \cdot Y_{P/S}=D\cdot \frac{flux\left(P\right)}{D\cdot c_{s}}=\frac{flux\left(P\right)}{c_{s}}=\frac{VP\left(N\right)}{c_{s}}$$ (9)

Thus, the volumetric productivity is equal to the productivity on substrate times the concentration of substrate in the medium. Therefore, for a given fixed concentration of substrate in the medium, *c*_*s*_, optimizing the model to maximize productivity on substrate is equivalent to optimizing the model to maximize volumetric productivity. Moreover, for a given *c*_*s*_, the linear objective function *flux*(*P*) can be used to perform such an optimization. For the particular case of citramalate production, *flux*(*P*) is given by the value of *λ*[*t*_*cout*_] (see Figure 5), and hence, the objective function will be the maximization of *λ*[*t*_*cout*_].

#### 2.5.3 Fermentation Experiments

Continuous cultures were grown in a DASbox^®^ Mini Bioreactor System (Eppendorf, Stevenage, UK). The *E. coli* strain used for all fermentation experiments was BW25113 Δ*ldhA* pET29a-*Cer*-BBaJ23119-RFP-*cimA3.7*. The *E. coli ldhA* deletion prevents lactate formation, improving flux towards citramalate (Webb et al. (2018)). The plasmid contains both the *cimA* gene, to enable citramalate production, and the *cer* gene to reduce loss of the plasmid through mis-segregation (Green et al. (2018)). Glucose-limited chemostat cultures (150 ml working volume) were grown at 37°C, with pH controlled to 7 and dissolved oxygen to >30% , in modified MS (Stephens and Dalton (1987)) medium (2 g *L* ^−1^ KH2PO4, 2  ml *L* ^−1^ trace metals solution (Vishniac and Santer (1957), 0.25 ml *L* ^−1^ antifoam polypropylene glycol, 4 g *L* ^−1^ NH4Cl, 0.4 g *L* ^−1^ MgSO4.7H2O). Biomass concentrations were determined by centrifuging measured samples from the fermenter into pre-weighed tubes, washing the pellets, and drying to constant weight at 100°C for 48 h. The supernatants from these samples were used to measure glucose, citramalate, and acetate concentrations. These analyses were performed using an UltiMate 3000 HPLC system (Thermo Fisher Scientific, Loughborough, UK) equipped with an Aminex HPX-87H ion-exclusion column (Bio-Rad, Hertfordshire, UK) and a RefractoMax520 RI detector (Knauer, Berlin, Germany). The mobile phase used was 0.1*%* (v/v) trifluoroacetic acid (TFA) in Milli-Q water.

## 3 Results

The validation of the model was carried out by comparing several simulation runs of the designed FN, see Figure 5, to previously obtained experimental results. These experiments were carried out under glucose-limited conditions, using three different concentrations of glucose in the supplied medium (5, 10 and 50 *gL* ^−1^), and different dilution rates.

Table 2 reports the numerical results obtained both *in vivo*, and *in silico* by the FN model. The columns of the table are divided in three parts: the first part sets the experimental parameters, i.e., the glucose concentration in the medium and the dilution rate for the glucose-limited continuous cultures; the second part reports the *in vivo* experimental results; and the third part reports the *in silico* results. For each pair of experimental parameters (glucose in medium and dilution rate), the columns corresponding to the “*In vivo* results” report the cell density (column “Biomass”), the concentration of citramalate (column “Citramalate in tank”), and the concentration of glucose (column “Residual glucose”) measured in the tank. In order to validate the model, the concentrations of citramalate and glucose in the tank are computed for each set of experimental parameters (glucose in medium and dilution rate) and measured biomass. The columns corresponding to the “*In silico* results” report the computed concentration of citramalate in the tank (column “Citramalate in tank”), the relative error of such predicted concentrations with respect to the measured *in vivo* concentrations (column “Citramalate relative error”), and the computed concentration of glucose in the tank (column “Residual glucose”).

**TABLE 2:** Data obtained after running the code that simulates the FN model implementing the experimental conditions and previous results.

Experimental parameters		*In vivo* results			*In silico* results		
**Glucose in medium (*gL* ^−1^)**	**Dilution rate (*h* ^−1^)**	**Biomass (*gDWL* ^−1^)**	**Citramalate in tank (*in vivo)* (*gL* ^−1^)**	**Residual glucose (*in vivo)* (*gL* ^−1^)**	**Citramalate in tank (*in silico)* (*gL* ^−1^)**	**Citramalate relative error (*%*)**	**Residual glucose (*in silico)* (*gL* ^−1^)**
5	0.1	1.88	1.79	0.0	1.32	35.60	0.0
10	0.03	3.22	2.49	0.0	1.87	33.16	0.0
10	0.1	3.41	3.09	0.0	3.46	−10.7	0.0
10	0.17	3.54	3.34	0.0	3.55	−5.92	0.0
10	0.23	3.55	3.32	0.0	3.67	−9.54	0.0
50	0.17	16.32	13.27	0.0	20.75	−36.05	0.0
50	0.23	16.86	13.11	0.0	20.22	−35.16	0.0

For a specific concentration of glucose and dilution rate, the biomass and the citramalate concentration reached the values showed in columns “Biomass” and “Citramalate in tank *in vivo*” of Table 2. From these results, it can be confirmed that the higher the concentration of glucose and dilution rate, the greater the amount of citramalate and biomass that will be produced.

The results obtained for a set of experiments (column “Citramalate in tank *in vivo*”) are consistent with the results obtained by the simulation of the FN model (column “Citramalate in tank *in silico*”) for the production of citramalate. The most similar outcome occurs when the glucose concentration was 10 *gL* ^−1^ as shown in the “Citramalate relative error” column in Table 2.

Notice that, in all cases, the supplied glucose is used up by the culture, i.e. the concentration of residual glucose is 0 *gL* ^−1^ (column “Residual glucose (*in vivo*)”). This fact is correctly predicted by the FN model (column “Residual glucose (*in silico*)”).

Once the model was validated, it was exploited to estimate the theoretical maximum productivity (see Subsection 2.5.2) of citramalate as well as the optimum biomass that produces its associated productivity.

**FIGURE 8:**
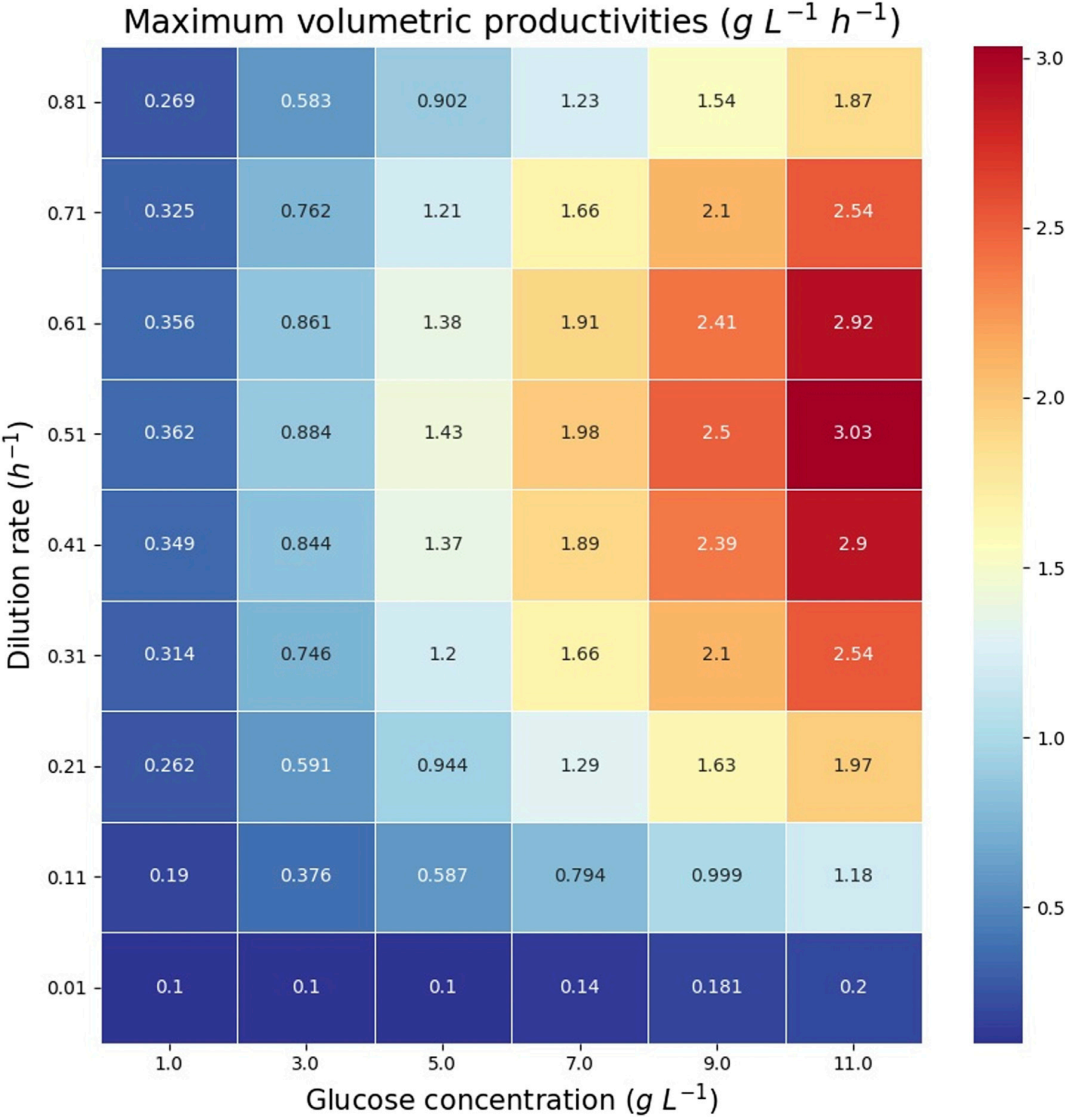
Heatmap reporting the maximum volumetric productivities (VPs) for each glucose concentration and dilution rate.

**FIGURE 9:**
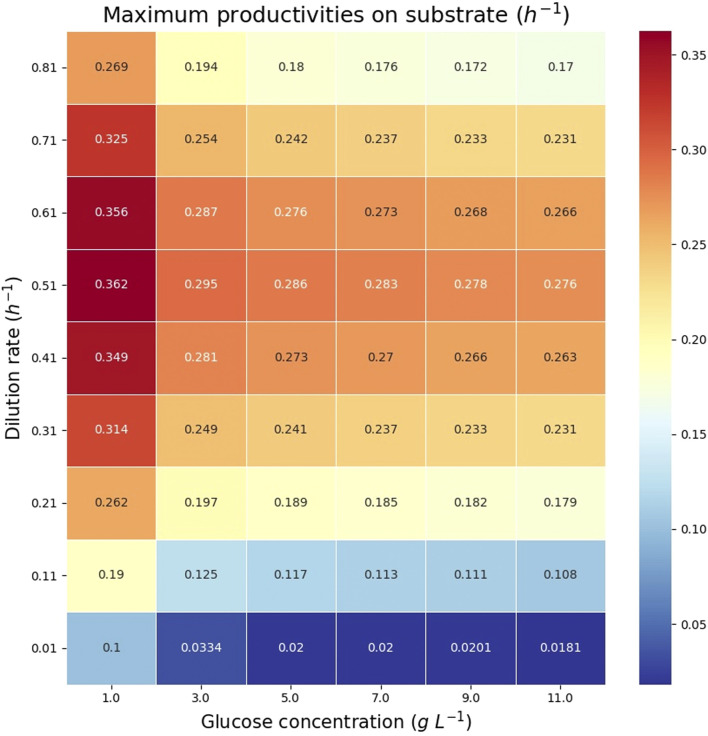
Heatmap reporting the maximum productivities on substrate (*PS*) for each glucose concentration and dilution rate.

**FIGURE 10:**
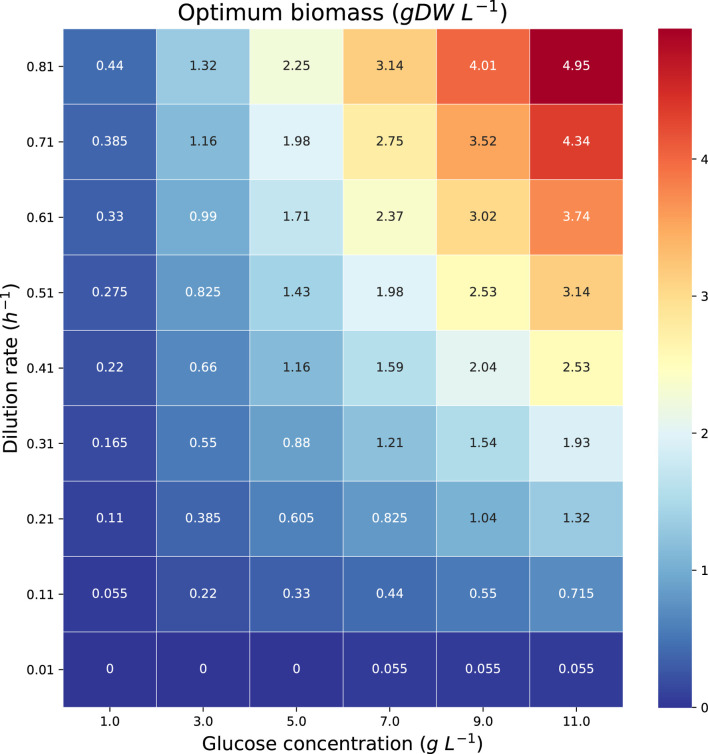
Heatmap reporting the optimum biomass concentrations associated with each of the maximum productivities in Figures 8, 9.

The theoretical maximum volumetric productivity (*VP*) and productivity on substrate (*PS*) of citramalate are reported in the heatmaps in Figure 8 and Figure 9, respectively. The cell densities, or biomass concentrations, for which the productivity is optimized are reported in the heatmap in Figure 10. For the explored glucose concentrations and dilution rates, the highest *VP* was reached when the glucose concentration was 11.0 *gL* ^−1^ and the dilution rate was 0.51 *h* ^−1^, such a productivity is obtained with a biomass of 3.14 *gDW L* ^−1^. With respect to *PS*, the highest value was obtained for a glucose concentration of 1.0 *gL* ^−1^ and a dilution rate of 0.51 *h* ^−1^, such a productivity is obtained with a biomass of 0.275 *gDW L* ^−1^. It is important to note that the maximum productivities for all concentrations of glucose are obtained when the dilution rate is 0.51 *h* ^−1^. As expected, the amount of biomass necessary to maximize the productivity increases as the dilution rate and the glucose concentration increase, Figure 10.

The reported *in silico* results were obtained by *fnyzer* (Júlvez and Oliver, 2020a) which transforms the FN in Figure 5 into a mixed-integer programming problem, and calls the CPLEX solver (IBM, 2010) to compute the numerical values.

## 4 Discussion

The mass production of commodity chemicals from fossil fuels can entail a serious negative impact in the environment. As a consequence, alternative approaches are being designed in order to redirect chemical production to more sustainable methods. However, the implementation of these novel approaches at an industrial scale requires optimization before they can replace traditional methods. To achieve this, biotechnology needs to exploit the advantages offered by computational models. Models can provide guidance for the design of experiments, give insights about the underlying mechanisms of the system, perform predictions and rule out infeasible hypotheses. Given the speed at which models can be simulated and optimized, they can save significant amounts of time, effort, and money in the wet lab.

In the last 2 decades, different modeling approaches have been developed, and particular attention has been paid to models of metabolism. In this work, it has been proven that the modeling formalism of FNs can integrate genome-scale constraint-based models, which lack detailed kinetic information, and kinetic models, which account for the concentration of the compounds of the system and are expressed as differential equations. Furthermore, FNs can also accommodate uncertainties inherent to the model, for example, partially unknown parameters.

An FN is represented as the combination of two nets: the event net and the intensity net. The event net models the stoichiometry, whilst the intensity net models the system dynamics. Such a graphical representation produces an overall view of the whole system. The analysis of an FN relies on the solution of a programming problem derived from the FN. If all the reaction rates are linear, i.e., the FN does not have guards, then the resulting programming problem includes only real variables with linear and quadratic constraints that can be solved very efficiently. As a consequence, FNs can handle efficiently genome-scale metabolic networks whose kinetic information is given by flux bounds and linear expressions that define the quantity of metabolites.

In contrast, if the reaction rates are not linear, they need to be approximated by piecewise linear functions, i.e. a guarded FN, which results in programming problems with real and binary variables. The complexity of the algorithms to solve mixed-integer programming problems is exponential in the number of binary variables. In order to obtain a balance between computational burden and accuracy of the model, the number of regions, and hence the number of binary variables, can be modified.

In Section 3, it was shown that FNs are a useful tool to predict the behavior of a complex system, such as a continuous culture in a bioreactor. The predictions of the citramalate production for a specific dilution rate and biomass were reliable in comparison to the experimental results obtained in the *in vivo* experiments, especially, the ones in which the glucose concentration in the medium was 10 *gL* ^−1^.

Although the simulations were highly predictive, the model could be improved further by adding some additional information, such as more components in the culture medium, or constraining the uptake of glucose depending on the glucose import rate, and similarly with the citramalate export rate. Both rates depend on protein transporters that can be saturated. Furthermore, the implementation of omics data could improve the model as well (Sánchez et al., 2017).

Not only was this method useful to reproduce the results of the *in vivo* experiments, but it can also help guide these experiments and optimize the conditions without wasting time and resources. The optimization performed in Section 3 showed that it is of special interest to explore *in vivo* the conditions that maximized the productivity in the computational simulations.

## Data Availability

The Python code generated for this work is available at: https://github.com/jlazaroibanez/opticomch.

## Footnotes

1 For clarity, it is assumed that if the labels and equations of a handler are omitted (see handlers *v*_*gfromtank*_ and *v*_*gtocell*_ in Figure 4), then an equality among all the connected elements holds. For instance if the labels *b*, *c*, *x* and the equation *b* = *c* = *x* are omitted in *v*_4_ in Figure 2, the implicit meaning is that the stoichiometric weights of all metabolites involved in reaction *R*_4_ are 1.

## Supplementary Material

Supplementary Data 1. Maximum_Productivities.xlsx: This spreadsheet reports the computed theoretical maximum productivities and their associated cell densities for dilution rates ranging in the interval [0.01, 0.81] h^−1^ and glucose concentrations in the medium ranging in the interval [1, 11] gL^−1^. 2. EcolicitFN.xlsx: This spreadsheet reports the fluxes and concentrations for a system with dilution rate D = 0.25 h^−1^, glucose concentration in the medium glc = 7.0 gL^−1^ and cell density X = 0.5 gDWL^−1^. For clarity, only positive fluxes are reported and reactions with null flux are omitted. 3. Regions_and_Runtime.xlsx: This spreadsheet reports the maximum productivity on substrate (PS), optimum biomass, and run-time for a dilution rate of 0.23 *h* ^−1^, a glucose concentration of 10 gL^−1^, and a number of regions in the interval [10, 200]. All the simulation results were obtained on a 4 x Intel i5-7200 CPU, 2.50 GHz running Ubuntu 19.10 with 7.7 GB of RAM.
